# The Study of Effects of Monophenolic Antioxidants, Sodium Anphen and Potassium Phenosan, on Cell Apoptosis by Fluorescence and Confocal Microscopy

**DOI:** 10.3390/ijms27083514

**Published:** 2026-04-14

**Authors:** Elena M. Mil, Anastasia A. Albantova, Ludmila I. Matienko, Maksim A. Korovin, Varvara V. Kuvyrkova, Alexander N. Goloshchapov

**Affiliations:** N. M. Emanuel Institute of Biochemical Physics, Russian Academy of Sciences, Str. Kosygina 4, 119334 Moscow, Russia; elenamil2004@mail.ru (E.M.M.);

**Keywords:** fluorescence and confocal microscopy, antioxidants, sodium anphene (ANa), potassium phenosan (PhK), splenocytes, Lewis carcinoma cells, apoptosis, Bcl-2 protein

## Abstract

Currently, poly- and monophenol antioxidants should be considered not only as inhibitors that interact with free radicals, but also take into account that they are biologically active substances that affect specific targets in cells and can induce the activity of certain genes or stimulate various signaling pathways. The phenols can directly influence different points of the apoptotic process, and/or the expression of regulatory proteins. In our present study the effect of two antioxidants, sterically hindered monophenols sodium anphene (ANa) and potassium phenosan (PhK), on cell apoptosis of splenocytes was studied by fluorescence and confocal microscopy. PhK has already been introduced into medical practice in the Russian Federation because it proved effective as an anticonvulsant and was useful in treating neonatal hypoxia. The study of ANa continues; it may be a promising anticancer drug for some types of tumors. The fluorescent and confocal microscopy methods demonstrate that ANa in combination with H_2_O_2_ enhances apoptosis in suspension of Lewis carcinoma cells and to a lesser extent in splenocyte culture. We also discovered that autofluorescence of FAD and immunofluorescence of NADPH enzymatic complexes (with the AV-FITC fluorophore) in splenocytes of normal cells increases symbatically. The autofluorescence of FAD in splenocytes of Lewis carcinoma cells significantly exceeded that of splenocytes of healthy animals. The exact distinctive result was obtained when using potassium phenozan. It turned out that PhK prevents the development of apoptosis in mouse splenocyte cell culture (F1(CBA×C57B)). The combined use of ANa and PhK had no effect on splenocyte apoptosis. We show that fluorescence and confocal microscopy allow observing and quantifying the apoptotic effect of ANa and hydrogen peroxide, and make it possible to visualize metabolic changes in the cell, increased FAD fluorescence in tumor cells and NADPH -oxidase complexes in splenocytes. The data obtained indicate the possibility of using ANa in combination with hydrogen peroxide as an antitumor drug acting on certain types of cells. The different effects of sterically hindered monophenols ANa and PhK on the level of the anti-apoptotic protein Bcl-2 in the cell were established. ANa acts to lower Bcl-2 levels, signaling apoptosis, while PhK prevents the development of apoptosis and induces repair processes.

## 1. Introduction

Apoptosis is a genetically controlled and evolutionarily conserved form of cell death of critical importance for normal embryonic development and for the maintenance of tissue homeostasis in the adult organism [1]. Cancer cells are characterized by a deregulated proliferation, and/or an inability to undergo programmed cell death. Research shows that phenols can exert chemopreventive effects towards different organ-specific cancers, affecting the overall process of carcinogenesis by several mechanisms: inhibition of DNA synthesis, modulation of ROS production, regulation of cell cycle arrest, and modulation of survival/proliferation pathways. In addition, phenols can directly influence different points of the apoptotic process, and/or the expression of regulatory proteins [1].

The Bcl-2 family of anti-apoptotic proteins plays a pivotal role in regulating apoptosis and cellular homeostasis, making them critical therapeutic targets in cancer and other diseases characterized by pathological cell survival. BH3 mimetics, small molecules that selectively inhibit anti-apoptotic Bcl-2 family members, have achieved significant clinical success, particularly in hematologic malignancies. However, several challenges remain, including resistance mechanisms, toxicity (such as MCL1 inhibitor-associated cardiotoxicity), and the intricate balance between apoptotic and non-apoptotic functions [2].

Natural and synthetic antioxidants, poly- and monophenols, and vitamins that are widely used in the treatment and prevention of various diseases, and in cancer chemotherapy, have recently been reconsidered to have unpredictable effects on the body [3]. Thus, it was found that when using vitamin E and some antioxidants, there was no effect on the growth of lung cancer tumors in mice, but active metastasis occurred [4]. In [2], beyond oncology, it highlighted the expanding therapeutic potential of BH3 mimetics in autoimmune, fibrotic, and infectious diseases, as well as regenerative and anti-aging medicine.

It was established that molecule polyphenols, naturally occurring compounds, exert anticancer effects by targeting different checkpoints in malignant cells and have a high specificity for inducing cell cycle arrest, autophagy, and apoptosis. They exert these anticancer effects by inhibiting telomere expression, angiogenesis, and metastasis, in addition to lowering the expression of transcription factors that regulate the expression of cytoprotective genes, lowering p53 activation, reducing Bcl-2 expression and mitochondrial membrane potential, and decreasing the expression of HIF-1α while increasing cellular apoptosis via downregulation of p-Akt expression [5].

**Figure 1:**
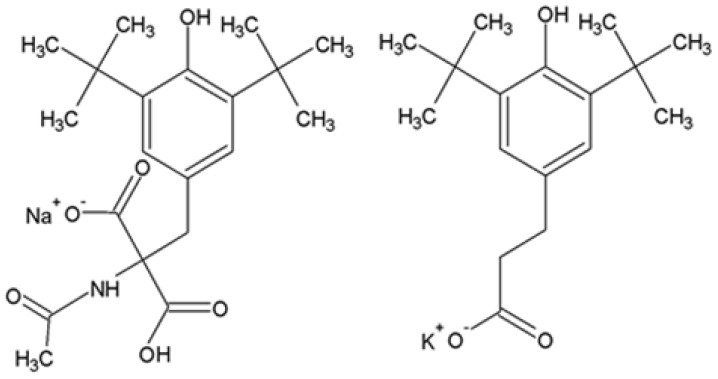
Antioxidants: Structural formulas of ANa and PhK.

The monophenols ANa and PhK (Figure 1) were synthesized for the first time and studied for a long time by a group of scientists from the school of Academician N.M. Emanuel at the Institute of Chemical Physics of Academician N.N. Semenov (RAS), later and now at the Emanuel Institute of Biochemical Physics of the Russian Academy of Sciences [6]. These antioxidants are derivatives of dibunol and belong to the class of sterically hindered monophenol compounds. It has previously been established that these antioxidants have anti-inflammatory properties, inhibit free radical oxidations, and interact with peroxide radicals [6,7,8]. In vivo studies on animals showed that the reaction rate constants of PhK and ANa with RO_2_• radicals (K_7_) are significantly lower than those of α-tocopherol and are comparable to the K_7_ rate constant for dibunol [6,7].

In medicine and the food industry, natural, non-toxic antioxidants were initially used: α-tocopherol, cysteine, glutathione, and thiourea. Later, artificial, synthetic, fat-soluble antioxidants—aromatic phenols—were tested. Dibunol (Di-tert-butyl phenol) was the first drug in this group of antioxidants. Ionol and dibunol were researched as antioxidants for the study of the mechanism of liquid-phased hydrocarbon oxidations. In medicine, dibunol has proven itself well in the treatment of burns, bladder cancer, ulcerative lesions of the skin, and mucous membranes [7].

It turned out that in the body, antioxidants affect various signaling systems leading to apoptosis or cell repair, and influence the level of the mitochondrial anti-apoptotic protein Bcl-2 [9]. When acting on cells, they exhibit various biological activities and can exhibit anticancer activity [7]. ANa is of practical interest because it has an antitumor effect, stopping the development of transplantable sarcoma 37 tumors in mice [7] and reducing the level of the anti-apoptotic protein Bcl-2 during the development of Lewis carcinoma in mice [9].

The aim of this study was with fluorescence and confocal microscopy to investigate the bioactivity of structurally similar AOs monophenols ANa and PhK, as well as their combination, namely to determine their impact on the signaling mechanisms of normal and tumor cells using fluorescence and confocal microscopy (Karl Zeic, Jena, Germany).

The fluorescence microscopy method is widely used in biochemical research and molecular biology. Since biological tissues fluoresce very weakly, the method involves the use of fluorophores. It is important to note that the method has low resolution compared to atomic force microscopy [10] and electron microscopy. The great advantage of the fluorescence microscopy method is the study of living cells directly during the experiment, which allows one to obtain much more information about metabolism [11].

## 2. Results

### 2.1. The Effect of Anphen Sodium (ANa)

Apoptosis in Lewis carcinoma cells was observed by exposure of phosphotidylserine to the outer cytoplasmic membrane (Fluorophore–Annexin V-FITC, fluorescence microscopy) [12].

When studying the concentration dependence of monophenols, it was found that the concentration of 5·10^−4^ M prevents the transplantation of Lewis carcinoma cells, and also quickly causes apoptosis of most cells of the Lewis carcinoma tumor cell culture within 2–3 h. A hydrogen peroxide concentration of 5 μM was used in the study, since concentrations (10 times greater) cause oxidative stress (see below).

**Figure 2:**
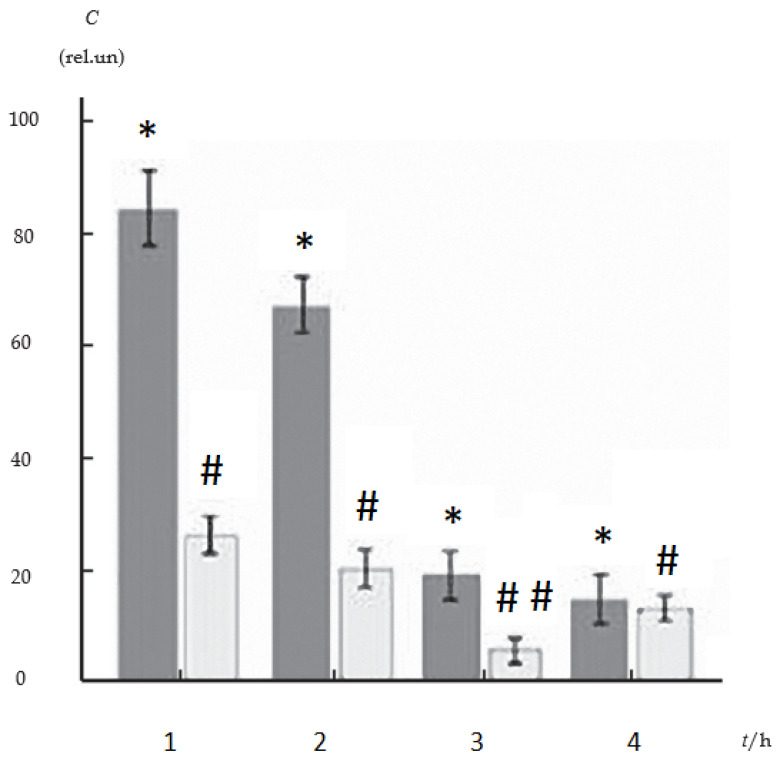
The content of two forms of Bcl-2 protein depending on time in Lewis carcinoma cells during incubation with ANa (5·10^–4^ M) (Bcl-2 homodimers-dark columns, monomers-light columns). Designations *,# refer to *p* < 0.05, ## refer to *p* < 0.01.

In our previous works by the immunoblotting method, we showed that with the introduction of the monophenol antioxidant ANa into a suspension of Lewis carcinoma tumor cells, there is a sharp decrease in the content of monomer and homodimer of the anti-apoptotic protein Bcl-2 (Figure 2) [13]. As can be seen from Figure 2, there is a decrease in protein content in the cell culture over time.

**Figure 3:**
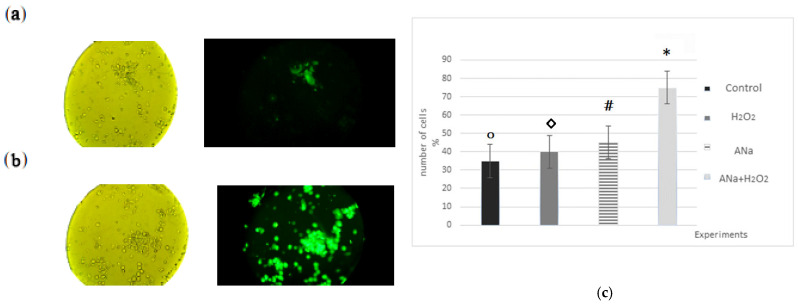
Microphotographs of Lewis carcinoma tumor cell suspension with Annexin V-FITC fluorophore: (**a**)—Control. 450 nm laser irradiation without additives; (**b**) Effect ANa = 5·10^−4^ M and hydrogen peroxide (5 µM) at 450 nm irradiation. The magnification is 100·4. (Splenocyte cells measure 6–8 µm). (**c**) Diagram of the increase in the percentage of apoptotic cells in Lewis carcinoma suspension, depending on the ANa (5·10^−4^ M), H_2_O_2_ (5 μM), {ANa (5·10^−4^ M) + H_2_O_2_ (5 μM)}. Designations *,#,◊,○ refer to *p* < 0.05.

Apoptosis in Lewis carcinoma cells was observed by exposure of phosphotidylserine to the outer cytoplasmic membrane (Fluorophore–Annexin V-FITC, fluorescence microscopy) [13]. As shown by fluorescence analysis, the number of apoptotic cells increased sharply 1 h after exposure to ANa. Preliminary administration of 5 µmol·L^–1^ hydrogen peroxide increased the permeability of cell membranes to ANa and the number of apoptotic cells (Figure 3). The onset of apoptosis, recorded by fluorophores, is comparable in time to the onset of a decrease in the level of Bcl-2 protein. We hypothesized that the mechanism of action of ANa may be associated with the interaction of the drug with the hydrophobic region (BH3) of proteins of the Bcl-2 family [12].

In this article, we show that fluorescence and confocal microscopy allow us to observe and quantify the apoptotic effect of ANa and hydrogen peroxide, and also make it possible to visualize metabolic changes in the cell, increased FAD fluorescence in tumor cells, and NADPH -oxidase complexes in splenocytes.

A confocal microscope makes it possible to additionally see the surface of the cell. Thus, we found that during apoptosis, not only is phosphatidylserine released to the cell surface, but also NOX complexes are exposed.

The study of the effectiveness of phenolic antioxidants was carried out on a suspension of mouse splenocytes and on a Lewis carcinoma cell culture.

**Figure 4:**
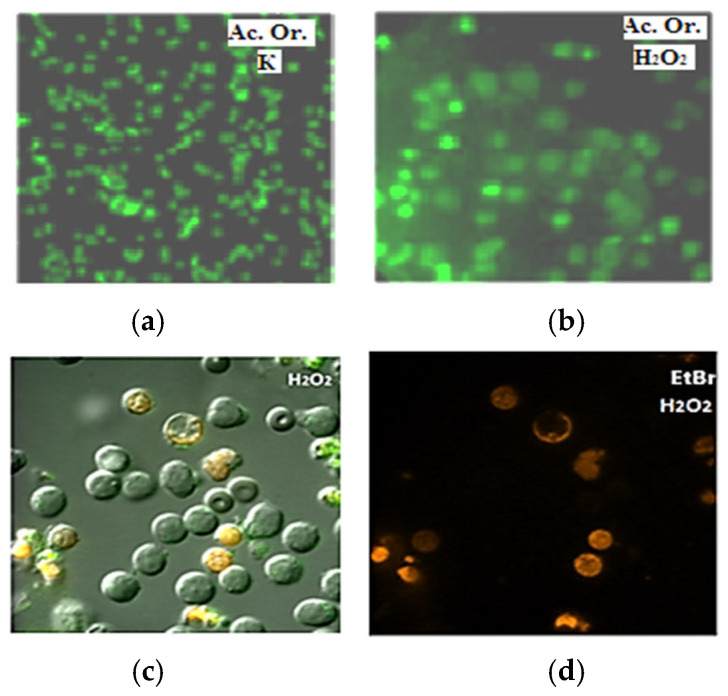
Splenocyte cells: (**a**,**b**)—live, native: stained with acridine orange (AcOr-green color): (**a**)—control, (**b**)—action of H_2_O_2_ (5 μM). (**c**)—light image of the same cells as (**d**)—identification of non-viable, dead cells with ethidium bromide (EtBr—red light)—action of H_2_O_2_. (**c**)—visible light illumination, fluorescence microscope. The magnification is: (**a**,**b**)—100·4; (**c**,**d**)—100·7. (Splenocyte cells measure 6–8 µm).

Using the fluorescence method (Figure 4), images of spleen cells splenocytes were obtained in control and with hydrogen peroxide. Light images of splenocytes show that the cells were mainly round in shape (95%), and some of the cells (less than 2%) consisted of flattened, possibly older cells.

**Figure 5:**
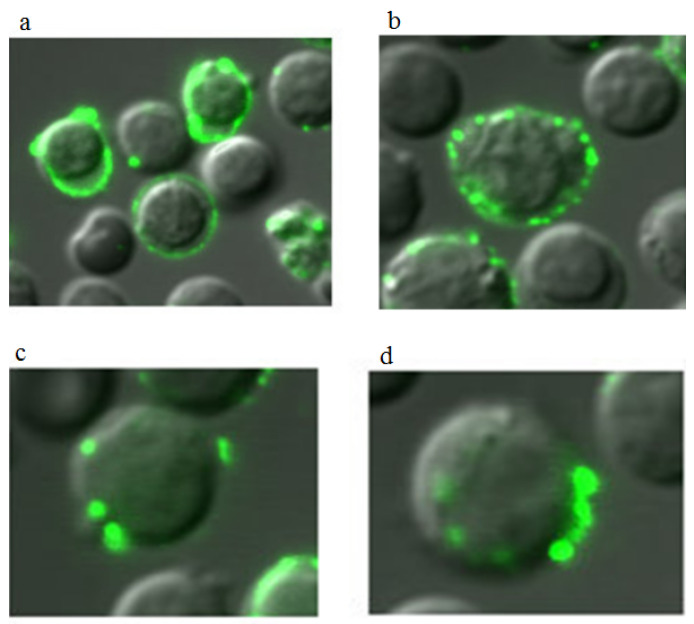
(**a**,**b**)—Splenocyte apoptosis induced by ANa. The AV-FITC fluorophore indicates the release of phosphatidylserine onto the outer cell membrane. Rounded structures of NAD(P)H oxidase enzymatic complexes are visible under a confocal microscope; (**c**,**d**) is FAD autofluorescence, without fluorophore, under 450-nanometer laser irradiation and a 480-nanometer filter. Bright luminescent spots. Fluorescence microscope. The magnification is: (**a**)—100·10), (**b**)—(100·10) and additional photo enlargement 1.5 times; (**c**,**d**)—100·12. (Splenocyte cells measure 6–8 µm).

It should be noted that in splenocytes (Figure 5), one can observe the intrinsic fluorescence of centers, such as the cofactor FAD in the oxidized state (450 nm light, 530 filter). When the fluorophore AV–FITC is added during cell apoptosis caused by ANa, immunofluorescence of the membrane is observed, and round structures of NAD(P)H oxidase enzymatic complexes can be observed on the outer membrane. NAD(P)H oxidases are complex membrane proteins containing the cofactors NADH and FAD, which are involved in redox processes [12,13,14,15,16,17].

The number of objects with the FAD cofactor in one cell changed from 1–2 in the control to 8–10 during the development of apoptosis caused by ANa and hydrogen peroxide. A number of altered cells (less than 1%) had a blurred glow (a diffuse fluorescence), which perhaps should be attributed to lipofuscin formations [14,16,18].

**Figure 6:**
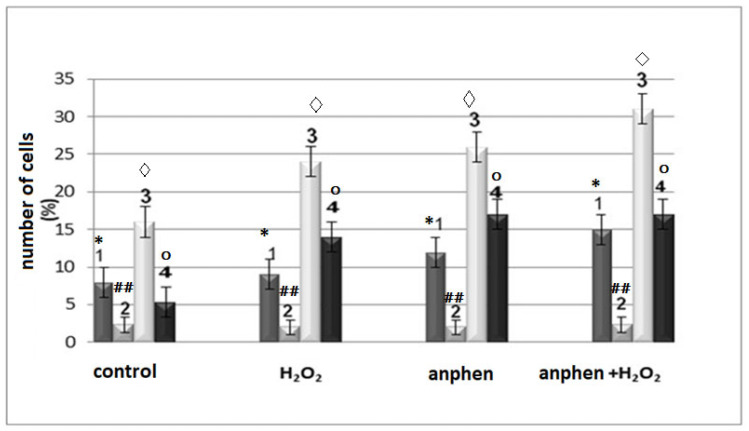
Diagram of changes in the number of apoptotic cells (splenocytes) and non-viable cells in the control group and under the influence of ANa (5·10^−4^ M), H_2_O_2_ (5 μM), and {ANa (5·10^−4^ M) + H_2_O_2_ (5 μM)}, recorded by various methods: 1—percentage of apoptotic cells (AV-FITC), 2—percentage of non-viable cells with ethidium bromide, 3—number of cells with FAD autofluorescence, 4—percentage of cells with NAD(P)H oxidase complex. The effect on the cell suspension was carried out for 2 h in medium 199 at 37 °C. Designations *,◊,○ refer to *p* < 0.05. Designations ## refer to *p* < 0.01.

Using a complex of EtBr with DNA (Figure 4, red), the number of living and damaged cells was determined. Thus, in Figure 6, column 2 in the control, the number of non-viable cells is 2–3%, under the action of ANa, and under the action of ANa together with hydrogen peroxide, the number of such cells increased to 6%. At the same time, the number of cells with apoptosis (determined with AV-FITC) in the splenocyte suspension in the control was 2–4% and increased after incubation with Anphen.

Figure 6 shows the number of cells with apoptosis, determined with AV-FITC-1 and non-viable cells (EtBr)-2 (see method). In the control, it was under the influence of ANa, hydrogen peroxide, and under combined exposure. An increase in the number of cells with FAD ox (3) and cells with NAD(P)H oxidase complexes was also shown. The exposure time of the drugs and control was 2 h. An increase in the fluorescence intensity of FAD correlated with an increase in the fluorescence of NADPH complexes, which suggests that FAD belongs to this complex. The luminescence of the cofactor [15,18] is also observed in other systems, but, in particular, in mitochondria, proteins of the electron transport chain extinguish it.

It was found (AV-FITC fluorophore) that ANa (5·10^−4^ M) activates apoptosis in splenocytes of white outbred mice: the number of apoptotic cells increases to 12% from 2–4% in the control after 2 h of exposure, and with the combined administration of ANa and hydrogen peroxide, the number of apoptotic cells increased to 15%. It is likely that in splenocytes, a 2 h exposure causes early cell apoptosis, since the number of dead cells with extranuclear DNA (EtBr) changed slightly (from 2% to 6%).

The process of apoptosis, as is known, includes the formation of complex structures, apoptosomes, which contain seven identical protein components and caspases, because of which the cell contents are converted into apoptotic bodies.

**Figure 7:**
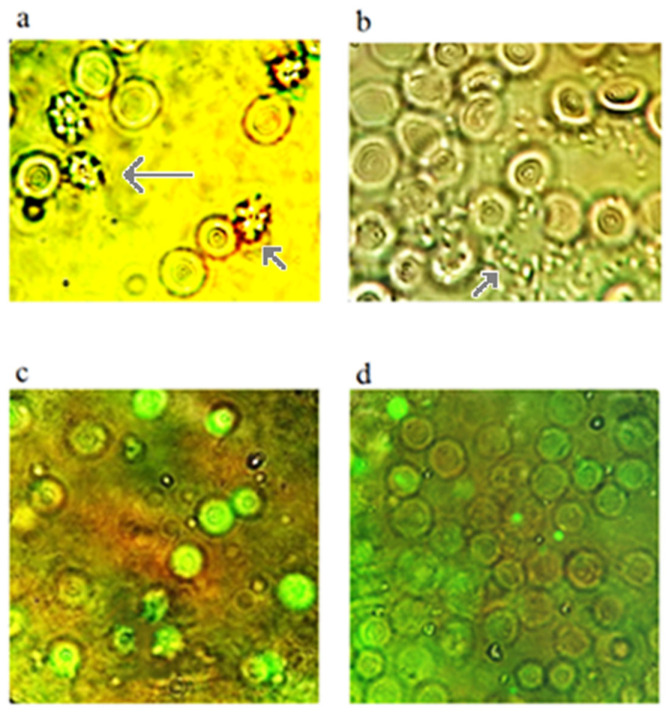
Images of splenocyte cells during the development of apoptosis under the influence of ANa (2 h): (**a**)—apoptosomes are visible (marked with arrows), (**b**)—rupture of the cell membrane and release of apoptotic bodies (marked with arrow). The comparison with the effect of PhK: (**c**)—solution of Triton X 100 for 2 h on splenocytes in the absence of PhK, (**d**)—the effect of PhK on the system with Triton X 100 for the same time (2 h). The magnification is: (**a**–**d**)—100·7. (Splenocyte cells measure 6–8 µm).

Figure 7a,b show images of splenocyte cells during the development of apoptosis (AV-FITC) caused by ANa. In cells in which apoptosomes have formed, apoptotic body production is visible. When this process is completed, the membrane of the enlarged cell ruptures. The apoptotic bodies are released into the intercellular space.

We previously found that ANa induces rapid apoptosis in Lewis carcinoma tumor cells (AV-FITC). The percentage of apoptotic cells reached 85–95% (after 2 h). When ANa and hydrogen peroxide were administered together, apoptosis was observed in the majority of cells. A number of studies have shown that low doses of hydrogen peroxide do not cause oxidative stress [18] and have a number of positive properties [13], but at the same time are capable of inducing apoptosis [17]. It has been shown that in lung cancer cells, H_2_O_2_ (50 μM) causes mitochondrial-type apoptosis by reducing the level of Bcl-2 and increasing the activity of caspases, and in lymphocytes it stimulates the extrinsic pathway of apoptosis through MAP kinase cascades [19]. It has also been shown that H_2_O_2_ affects tyrosine phosphatases and stimulates the formation of NADPH oxidase complexes.

Thus, ANa in combination with H_2_O_2_ (Figure 6 and Figure 7) stimulated apoptosis of both normal and tumor cells. At the same time, ANa is a completely non-toxic compound [7], which can serve as a useful antioxidant property, as a promising antitumor compound.

### 2.2. The Effect of Potassium Phenosan

The action of the antioxidant phenosan potassium, similar in structure to sodium anphen, revealed a number of positive properties: antimicrobial and anti-inflammatory, as well as radioprotective effects when irradiated in sublethal doses; it also prevented severe complications in the event of heart attacks and strokes [20,21,22,23].

**Figure 8:**
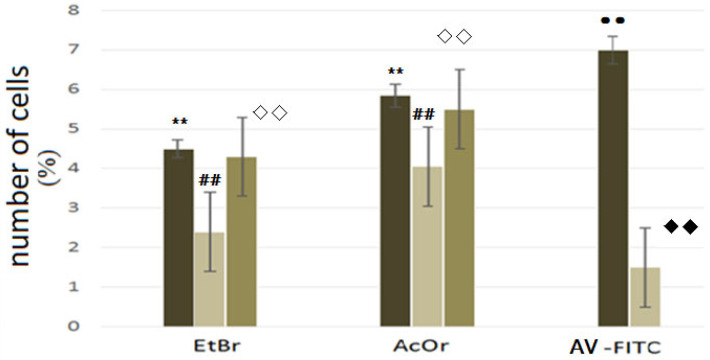
Ehe effect of PhK (2), H_2_O_2_ (3) (control (1)) on cells (first 2 groups experience). A separate group of experiments with the addition of Triton X 100 (fluorophore AV-FITC): (1) control-splenocyte cell culture with the addition of Triton X 100; (2) introduction of phenozan + Triton X 100. The *Y*-axis shows the percentage of cells exposed to the dyes. The *X*-axis shows the fluorophores: EtBr, AcOr, and AV-FITC. Designations **, ##, ◊◊, ●●, ♦♦ refer to *p* < 0.01.

As mentioned earlier cell apoptosis does not occur after 2 h of incubation of cells with K- phenosan (5·10^−4^ M) (Figure 7c,d). Figure 8 shows number of stained cells under the action of PhK and hydrogen peroxide. It shows the quantitative content of splenocyte cells with the fluorophores AV-FITC, AcOr and EtBr during cultivation for 2 h at 37 °C in medium 199, with the addition of 0.1% Triton X 100.

We found (EtBr dye) that exposure to potassium phenosan (5·10^−4^ M) reduced the number of non-viable cells relative to the control 2 h after administration, which is consistent with its repair properties [23]. In a study with the AcOr dye, it was shown that PhK had almost no effect on cell permeability, but with the introduction of H_2_O_2_ (5 μM), the permeability increased. In a study with the AV-FITC fluorophore in the presence of Triton X 100, it was shown that the introduction of PhK reduced the number of cells developing apoptosis in the suspension, probably because this process was at a reversible stage.

When phenosan K and anphen Na, which cause different effects in the cell, were jointly introduced into a suspension, no apoptosis was observed.

## 3. Discussion

The non-toxicity of the artificial monophenols under consideration was established at a specialized Scientific Center for the Study of the Safety of Bio Active Substances (SCBAS) (Moscow). PhK was introduced into medical practice due to its proven effectiveness as an anticonvulsant and its usefulness in treating neonatal hypoxia. PhLi (Li-Phenosan) has found application in medical practice as an antiepileptic drug in Russian Federation. ANa is still being studied, including as an antitumor drug, as it effectively inhibited the development of sarcoma 37 in mice [4].

One of the strategies for antitumor therapy is the search for drugs and the creation of new substances with targeted effects, including those that trigger the process of apoptosis along the mitochondrial pathway. It turned out that sodium anphene from the class of sterically hindered phenols in Lewis carcinoma cells is able to influence anti-apoptotic proteins of the Bcl-2 family, reducing their content, which indicated the onset of apoptosis along the mitochondrial pathway [12,23]. The sterically hindered phenol phenosan potassium, which is similar in structure, does not have such properties. It can be assumed that the opposite activity of the studied antioxidants as antitumor drugs may be associated with the peculiarities of their structure. In [24], the authors utilized atomic force microscopy (AFM) to evaluate the effects of polyphenols on the BAX/Bcl-2 binding mechanism. They demonstrated at the molecular scale that polyphenols quantitatively affect the interaction forces, kinetics, thermodynamics, and structural properties of BAX/Bcl-2 complex formation. Combined with surface free energy and molecular docking, the results revealed that polyphenols are driven by multiple forces that affect the orientation freedom of PPIs (protein–protein interactions), with hydrogen bonding, hydrophobic interactions, and van der Waals forces being the major contributors [24]. One possible explanation for the regulatory effect of monophenols on apoptosis is associated with their hydrophobic properties. Phenols can occupy the hydrophobic pocket of Bcl-2, mimicking the BH3 domain of proapoptotic activator proteins. It is possible that the hydrophobic fragment in the para position to the OH group of ANa (with hydrophobic -CH_3_ methyl group) promotes the binding of this antioxidant to the receptor site of the hydrophobic pocket of proteins of the Bcl-2 family, which regulate apoptosis. The presence of a secondary amino group and carbonyl can allow ANa to be integrated into the network of hydrogen bonds on the surface of the protein. Unlike ANa, PhK prevents the development of apoptosis and induces repair processes.

Mitochondrial apoptosis is associated with proteins of the Bcl-2 family: Bcl-2, Bcl-xL and Mcl-1 [23]. At the same time, it has been established in a number of studies that changes in the level of the anti-apoptotic regulatory protein Bcl-2 play an important role and can predict the direction of development of processes in the cell (towards repair or apoptosis) [25,26,27,28,29,30,31,32,33].

It is known that in cancer cells, apoptosis along the mitochondrial pathway is significantly suppressed, firstly, due to a mutation of the p53 regulator protein, as well as an increase in the expression and stability of proteins of the Bcl-2 family [23,27]. One of the ways to treat cancer is to enhance the apoptosis of tumor cells by acting on proteins of this family. Sodium anphen can be used for these purposes.

In [28], based on HeLa Kyoto cells, it was suggested that the ratio of endogenous cofactors FAD and NAD(P)H can be considered as a metabolic characteristic of cancer diseases, and also makes it possible to evaluate the effect of anticancer drugs.

It has been established that the fluorescence method can detect apoptosis caused by ANa, including the percentage of cells in an apoptotic state with exposure to phosphatidylserine, enhanced autofluorescence by FADox and fluorescence of NAD(P)H oxidase complexes using the fluorophore AV-FITC (Amresco, Solon, OH, USA).

NADPH oxidase (NOX) complexes are found in virtually all mammalian cells. Their main function is the formation of superoxide anion or hydrogen peroxide. This system is used by the cell to protect against bacterial and microbial infection. It has been established that when this system is disrupted, a number of pathologies can occur, such as Alzheimer’s disease and other neurodegenerative diseases. It turned out that NOX expression is associated with neoplasms, including malignant ones [14,15,16].

**Figure 9:**
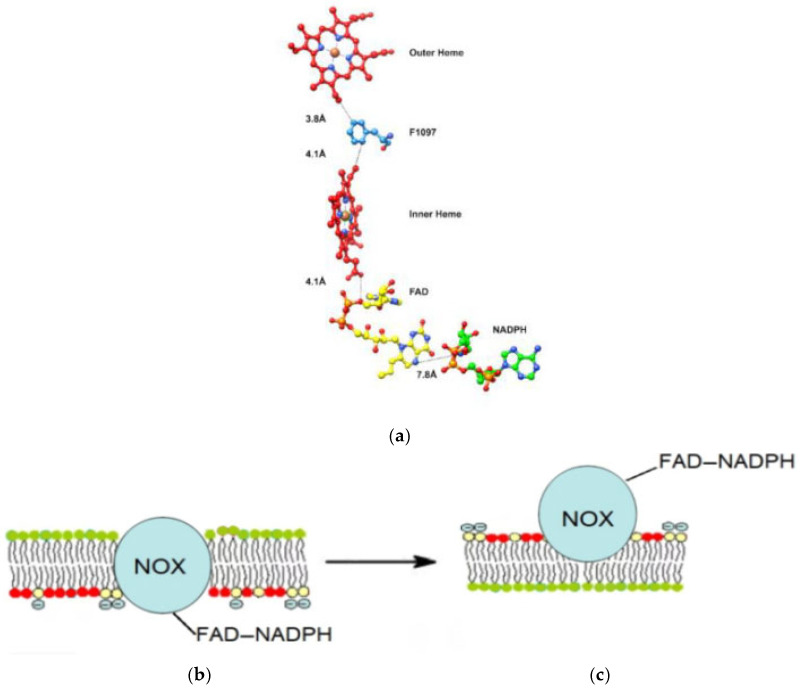
Proposed exposition of phosphatidylserine and NOX to the membrane surface during apoptosis. (**a**)—Structure of NADPH oxidase (NOX). The C-terminal region of the chain contains a redox pair of cofactors FAD and NADPH. The close location of FAD and NADPH in the active site of NADPH oxidase is visible [33]. (**b**)—pre-apoptotic state, (**c**)—apoptosis with exposure of phosphatidylserine and possible turnover of the complex.

It is known that NAD(P)H oxidases in the NOX family complex contain six transmembrane helices and five loops localized in the aqueous phase. In this case, the NAD(P)H and FAD centers in the C-terminal region are close to each other, for example [33] (Figure 9a).

These complexes arise during the process of apoptosis—in the first hours, brightly colored cell membranes are visible, indicating exposure to phosphatidylserine, and then rounded structures of enzymatic NADPH oxidase complexes appear (Figure 5a,b). We hypothesized that during the process of apoptosis, the C-terminal region of FAD and NADH of the NAD(P)H oxidase protein may be released onto the cell membrane surface (Figure 9b,c).

We have shown that the apoptotic effect of anphen sodium is more pronounced in Lewis carcinoma cells than in normal splenocyte cells. FAD levels are also elevated in tumor cells.

## 4. Materials and Methods

1. Sodium Anphen and Potassium Phenosan

Antioxidants sodium anphen (ANa): 2-(carboxy)-2-(N-acetylamino)-3-(3′,5′-di-tert-butyl-4′-hydroxyphenyl)-sodium propionate and potassium phenosan (PhK): (1-(carboxy)-1-(N-methylamide)-2-(3′,5′-di-tert-butyl-4′-hydroxyphenyl)-potassium propionate (Figure 1) were synthesized at the Institute of Chemical Physics, like other drugs of the class of sterically hindered phenols [6]. The drugs were dissolved in physiological solution.

### Tumor Cells Splenocytes

The choice of model, based on Lewis lung cancer cells, is because these cells have the same characteristics as ordinary cells. Splenocytes are a convenient model of isolated cell culture.

The study used white outbred mice, as well as first-generation F1(CBA×C57B) hybrid mice (Nursery Stolbovaya, Pushchino, Moscow region) (3–4 months old, weighing 22–25 g), from which splenocytes were isolated. The mice were decapitated under ether anesthesia.

Lewis carcinoma cells of mice were isolated from the tumor on the 14th day after tumor cell transplantation (according to the method [7]). White mongrel mice from the Stolbovaya nursery were used. To obtain a suspension of cells, including tumor cells and splenic splenocyte cells, according to [9], the tumor was crushed in saline solution, dispersed repeatedly, and filtered through a nylon (100 microns) mesh. The cell suspension was precipitated at 150 g and the precipitate was resuspended to obtain a cell suspension. The cells were then injected into the thigh muscle of the hind leg (7·10^6^ cells/mouse) or used in in vitro experiments. The number of cells was determined using a Goryaev chamber (Minimed, Kostroma, Russia).

Cells in the presence of the studied compounds were incubated for 2 h at 37 °C in medium 199 on a solution of Henks (Institute of poliomyelitis and viral encephalitis of the Russian Academy of Sciences) under different conditions: in the control, with the addition of ANa (5·10^−4^ M), hydrogen peroxide (5 μM), and also with the sequential introduction of hydrogen peroxide (5 μM) and ANa (5·10^−4^ M). Similarly, PhK (5·10^−4^ M) was introduced.

2. Determination of Bcl-2 by immunoblotting [12].

The content of Bcl-2 protein in a suspension of Lewis carcinoma cells was determined using antibodies Monoclonal Anti-BCL-2 cloneab 323123 (Abcam, Cambridge, Cambridgeshire County, UK) to the synthetic region 50–150 of the Bcl-2 protein and a second antibody—horseradish peroxidase—labeled immunoglobulin anti-rabbit IgG (Sigma, St. Louis, MO, USA). The quantitative content of protein in the band was determined using the AEC Staining kit (Sigma). To do this, the stained blots were scanned and the optical density of the corresponding bands was calculated using the BMP ImageJ 1.54r programs.

3. Fluorescence microscopy

The samples were examined on a fluorescence microscope with excitation at 450 nm and a filter (530) that cut off the excitation light. The work was carried out on a CarlZeissJena laboratory microscope (Jena, Germany, Karl Zeic Jena fluorescence microscope with a 450 nm laser) equipped with optical excitation systems based on diode lasers and filtering, followed by recording on a web camera (PK 910H, New Taipei City, China). Splenocyte cells measure 6–8 µm. A fluorescence microscope provides magnification of 400–2000.

Using Image J, the total number of cells and those undergoing apoptosis were quantified.

AcOr and EtBr dyes were used; the latter allows recording DNA fluorescence of living, apoptotic and dead cells [9], which differ in luminescence in the green, orange and red regions, respectively. The AV-FITC fluorophore is an indicator of apoptosis; it binds to phosphatidylserine, which appears on the surface of the cell plasma membrane at the beginning of the apoptosis process.

4. Confocal microscopy.

The number of apoptotic cells in the suspension (in %) was determined by using AV-FITC (absorption maximum 488 nm) on a confocal microscope, compared with the total number of measured cells in the suspension (in light optical microscopy mode).

Confocal images were acquired using an AxioObserver Z1 microscope (Carl Zeiss, Jena, Germany) equipped with a Yokogawa confocal unit (CSU-X1; Yokogawa Corporation of America, Sugarland, TX, USA). Splenocyte cells measure 6–8 µm. A confocal microscope provides magnification of 1000 or more.

Statistical processing of experimental data was carried out using the Statistica 6 program with the determination of average values and 95% confidence intervals. We used Student’s *t*-test to determine statistically significant differences.

5. Reagents:

Aether for anesthesia (Medkhimprom, Balashicha, Moscow region, Russia), ethidium bromide (Amresco, Solon, OH, USA), acridine orange (Scharlab, Barcelona, Spain), AV-FITC, phosphate buffer (pH 7.3–7.5) (Amresco, Solon, OH, USA); bovine serum albumin BSA (Sigma, USA); HEPES (Biomedicals, Eschwege, Germany), medium 199 (Institute of Poliomyelitis of the Russian Academy of Sciences, Russia), and KCl (Serva, Heidelberg, Germany).

Medium 199: complex nutrient medium for culturing a wide range of animal and human cells. It contains a balanced composition of inorganic salts (Hanks or Earle), amino acids, vitamins, and glucose, phenol red, and specific components (adenine, adenosine, hypoxanthine, and thymine) on a solution of Henks (Institute of poliomyelitis and viral encephalitis of the Russian Academy of Sciences) and Triton X 100 (Serva, Heidelberg, Germany).

## 5. Conclusions

In our work, we investigated the effect of phenolic antioxidants on splenocytes. It has been shown that these cells are a convenient model for studying the processes of apoptosis and cell repair. It was found that ANa and PhK have opposite effects on cell metabolism.

The mechanism of action of PhK in small and ultra-low doses, causing repair effects, turned out to be multifaceted and may be associated with the activation of not only Bcl-2, but also Cu, Zn-SOD and protein kinase C. PhK is not only a biologically active substance, but also an active antioxidant that reduces lipid oxidation in mitochondria.

The property of anphen Na to induce cell apoptosis is probably mainly due to its effect on reducing the level of the anti-apoptotic protein Bcl-2, which leads to the development of apoptosis through the mitochondrial pathway.

Antioxidants, being a large group of biologically active substances, are of great interest to science and, in particular, medicine, but their role in the treatment of diseases remains for the most part the subject of scientific debate.

## Data Availability

All of the experimental data presented belong to the authors of this manuscript and are available.

## Abbreviations

Annexin V-FITC (AV-FITC), ethidium bromide (EtBr), acridine orange (AcOr), potassium phenosan (PhK), sodium anphen (ANa).
